# Scour ponds from unusually large tsunamis on a beach-ridge plain in eastern Hokkaido, Japan

**DOI:** 10.1038/s41598-023-30061-9

**Published:** 2023-02-21

**Authors:** Yuki Sawai, Toru Tamura, Yumi Shimada, Koichiro Tanigawa

**Affiliations:** 1 Geological Survey of Japan, National Institute of Advanced Industrial Science and Technology (AIST), Site C7, 1-1-1 Higashi, Tsukuba, Ibaraki 305-8567 Japan; 2 Graduate School of Frontier Sciences, The University of Tokyo, Chiba, 277-8563 Japan; 3 Institute for Space-Earth Environmental Research, Nagoya University, Aichi, 464-8601 Japan

**Keywords:** Natural hazards, Solid Earth sciences

## Abstract

Scour ponds from unusually large tsunamis cut across the crest of a beach ridge in Kiritappu marsh, eastern Hokkaido. No fewer than ten of these ponds were imaged by photogrammetry as elongate topographic depressions as large as 5 m by 30 m. Sediments in these ponds are underlain by unconformities that were detected with ground-penetrating radar and observed directly in cores and a slice sample. Sediment deposits in the ponds contain peat and volcanic ash layers, the ages of which suggest that the scouring occurred during tsunamis generated by spatially extensive thrust ruptures along the southern Kuril trench, most recently during the early seventeenth century and its predecessor during the thirteenth–fourteenth century. Some of the ponds appear to have been formed during one tsunami and refreshed during later successors. This evidence of recurrent erosion suggests that the shoreline may retreat as part of earthquake-related cycles of coastal uplift and subsidence.

## Introduction

**Figure 1:**
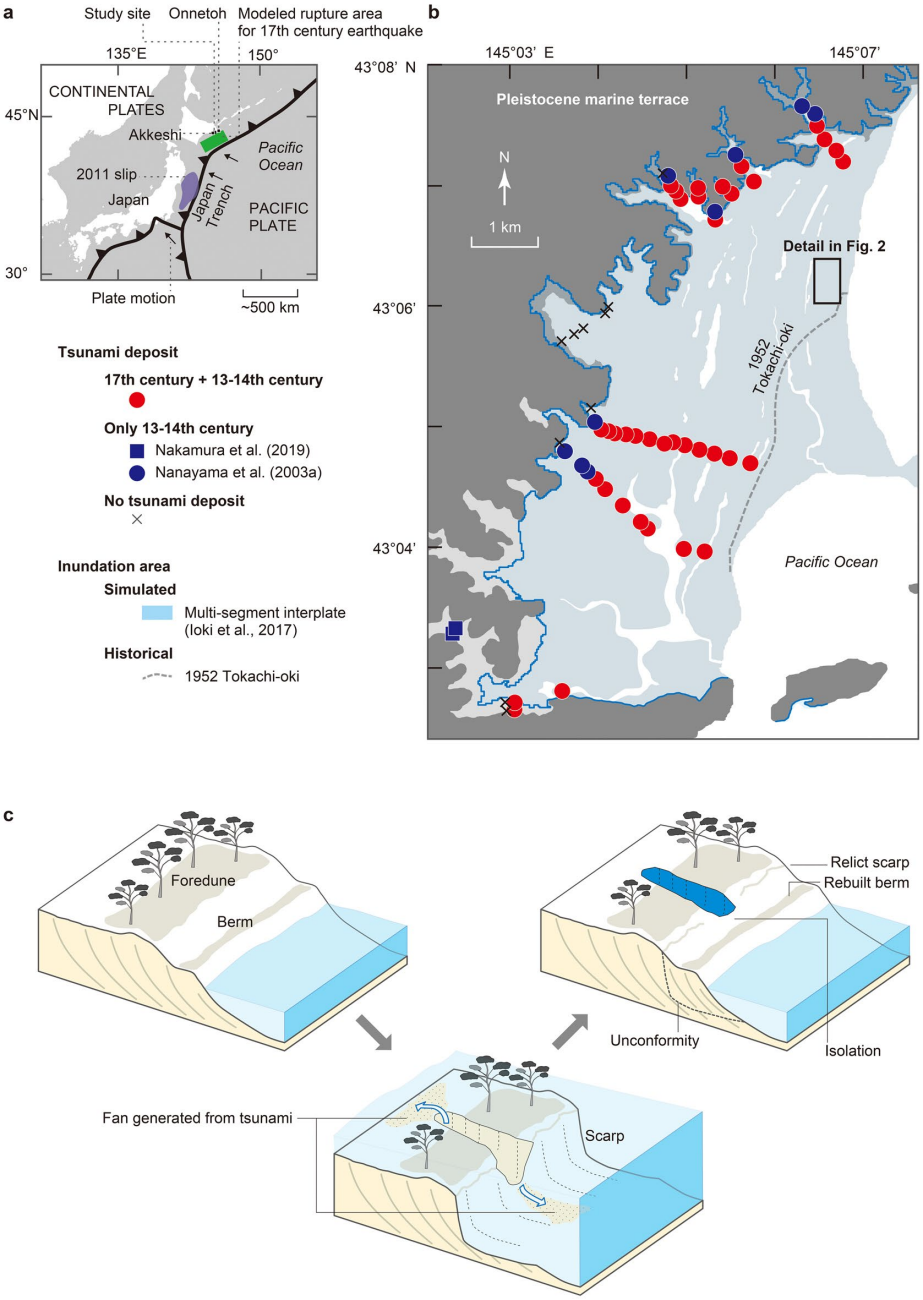
(**a**) Tectonic setting of Japan and location of the study site. The rupture of the 2011 earthquake is from Ozawa et al. (2011)^63^. (**b**) Map of the Kiritappu marsh showing distributions of tsunami deposits formed in the seventeenth century and thirteenth-fourteenth century^3,64^, historical tsunami inundation area^3,5^, simulated inundation area from a multi-segment interplate earthquake tsunami^3,5,7^, and Pleistocene marine terraces^65^. Inter-ridge ponds defined by beach ridges trend parallel to the present shoreline. (**c**) Cartoon showing the formation of a scour pond. The foredune and berm are reamed out perpendicular to the contemporary shoreline by inflow and outflow currents. The resulting scour pond then becomes isolated as the sandy beach is rebuilt with drift sand due to daily tides. The fan deposit created by inflow currents remains as a tsunami deposit upon the foredune and marsh. Generic Mapping Tools^66^ was partly used to create Fig. 1a. Figure 1b was modified from Sawai^1^.

Recent tsunami research in eastern Hokkaido, northern Japan, has produced new insights into the earthquake and tsunami history of the southern Kuril trench^1^. Official long-term forecasts of subduction zone earthquakes originating from the Kuril trench formerly relied only on historical records dating from the nineteenth century and recent instrumental observations^2^. However, sandy tsunami deposits beneath coastal marshes along the Pacific coast of Hokkaido have revealed that great tsunamis recurred a few centuries apart and caused inundations exceeding those of historical and recent tsunamis^3–6^ (Fig. 1a,b). Numerical simulations show that the sources of these tsunamis were multi-segment earthquakes having longer rupture lengths than those in the twentieth and twenty-first centuries in the southern Kuril trench^5–7^. The most recent and penultimate tsunamis are estimated to have occurred in the seventeenth century and the thirteenth to fourteenth century, respectively^3,4,8,9^. Fossil diatom assemblages in coastal sediments have revealed preseismic subsidence and > 1.5 m postseismic uplift before and after the seventeenth century event^10,11^.

Extreme tsunami waves produced by coseismic seafloor deformation propagate to surrounding coasts and erode the foredunes and sandy ridges of beaches, causing shoreline recession and leaving sculptural traces^12–21^. Along the Sendai plain, waves of the 2011 Tohoku tsunami produced funnel-like openings a few tens of meters wide on beaches and breached existing inlets perpendicular to the shoreline. Each breach became isolated from the sea in less than a year as the beaches were rebuilt, creating what we refer to here as scour ponds^1,12–15^ (Fig. 1c). Similar scour ponds and breaches were recognized after the 2004 Indian Ocean tsunami^16,17^ and the 1960 Chile tsunami^18–21^, as well as in the British Virgin Islands^22^. Such geomorphic features are expected to provide information on tsunami inundation and erosion from events preceding historical records, but identifying prehistoric scour ponds is not straightforward. In this study, we integrated unmanned aerial vehicle (UAV)-based photogrammetry, ground-penetrating radar (GPR) profiles, and sedimentological, paleontological and chronological analyses to identify scour ponds generated by prehistoric great tsunamis in eastern Hokkaido.

## Results

### Identifying trough-shaped depressions on relict beaches

We used structure-from-motion multi-view stereo photogrammetry, using a quadcopter UAV, to capture the topography of the study area. Although optical imagery generally reflects the surface of natural vegetation (e.g. trees with leaves and grasses), our survey was performed at a time of year between the late winter thaw and the germination and budding of plants (Supplementary Fig. S1). Therefore the resulting digital surface model (DSM) recorded the topography of the bare ground surface well. The elevation of the scanned area ranges from 13.7 m to − 3.38 m (relative to mean sea level at Tokyo Bay). The highest and lowest elevations represent anthropogenic modifications (e.g. houses) and marshy locations, respectively.

**Figure 2:**
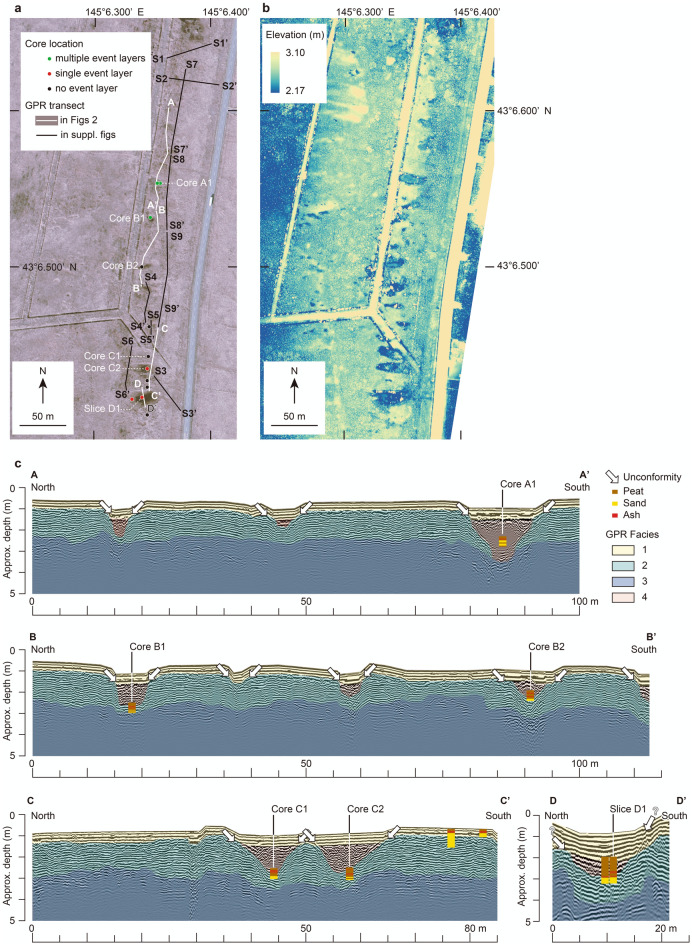
Details of the study site and GPR transects. (**a**) Aerial photograph (CHO-78–07-06B-0012, taken by Geospatial Information Authority of Japan) of the area outlined in Fig. 1b showing core locations and GPR transects. (**b**) DSM obtained in this study, with elevation range restricted to display surface features of the beach ridge. (**c**) Annotated GPR images along the transects shown in (**a**). The four GPR facies identified in this study are differentiated by color. Vertical exaggeration is approximately 4 × . The original GPR images are displayed in Supplementary Fig. S3.

We applied GIS software to the DSM to focus on elevations between 3.10 m and 2.17 m so as to accentuate the elevation range of the beach ridge and marshy places (Fig. 2b). One striking feature of the beach ridge was the crooked line of its crest, which traced the outlines of oval or funnel-like ponds with long axes perpendicular to the shoreline (Fig. 2b).

### Ground-penetrating radar

We assembled a GPR profile approximately 920 m long from a set of transects parallel (transects A–D and S4–S9) and perpendicular (transects S1–S3) to the shoreline (Fig. 2, Supplementary Figs. S2 and S3). The shore-parallel profiles included four radar facies (Fig. 2a and Supplementary Fig. S3). The first is ground surface reflections, consisting of a series of parallel reflections beneath the surface sediment that extended over all of the transects. The second facies, recognized beneath the first facies, consists of parallel or subparallel reflections that are interrupted by concave erosional surfaces near oval ponds (arrows in Fig. 2c). The third facies is a series of faint convex reflections below the second facies. The fourth facies consists of rough-textured wavy reflections filling the depressions in the second facies.

Similarly, the shore-perpendicular profiles have three facies, the first of which is ground surface reflections (Supplementary Fig. S2). The second facies, parallel or subparallel reflections, is interrupted near the seaward edge of the beach ridge (see arrow in Supplementary Fig. S2). The third facies, the deepest, consists of flat or seaward-dipping reflections.

### Stratigraphy

**Figure 3:**
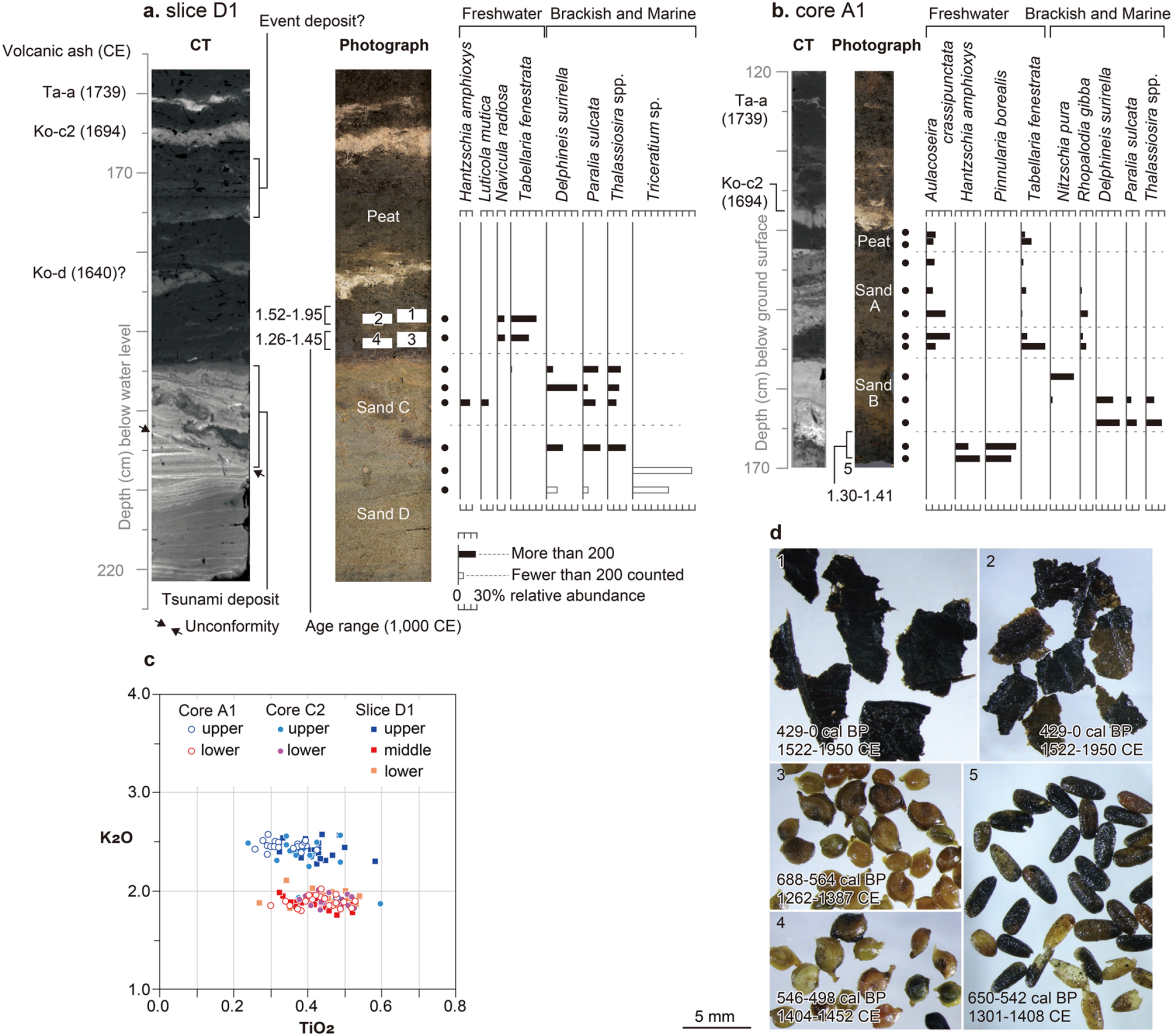
Examples of core samples taken in this study. (**a**) Slice D1. (**b**) Core A1. Shown for each are a CT image with ash layers and structures labeled (left), a photograph with sedimentary units labeled (middle), and changes in compositions of selected diatom taxa (right). White boxes 1–4 in the middle photograph of (**a**) show samples taken for radiocarbon dating. (**c**) Cross plots of K_2_O and TiO_2_ of glass shards from ash layers in selected cores. (**d**) Plant macrofossils from peat in slice D1 and core A1.

Sediment cores showed that the deposits beneath Kiritappu marsh consist of layers of peat, sand, and volcanic ash (Fig. 3). In beach ridge locations, an upper layer of peat 0.2 m thick grades downward into sand (sand D). This peat layer includes two or three volcanic ash layers. The underlying fine-grained sand is thick and has clear parallel lamination. In scour pond locations, the peat layer is thicker than elsewhere and contains as many as two interbeds of gravel-sandy layers (sands A and B). The upper layer (sand A) contains multiple grading upward from medium sand to fine-sandy mud, and the lower layer (sand B) grades upward from granules and coarse sand to fine sand (Fig. 3b). The boundary between peat and sand C in the scour ponds is consistent with the erosional surface apparent in GPR images.

The X-ray computed tomography (CT) images of core samples allowed us to observe detailed sedimentary structures in the sand layers (Fig. 3). In our sliced sample from a scour pond site (slice D1), the upper and lower parts of sand D contain clear parallel to subparallel lamination (Fig. 3a). The lamination is cut diagonally and overlain by chaotic sand (sand C). The chaotic sand contains mud clasts and wavy lamination at its top. These structures are only recognizable in CT images. Similarly, the CT image showed density differences in the sediments just below the middle volcanic ash (Fig. 3a) that were not otherwise apparent.

Fossil diatom assemblages and plant macrofossils are evidence of past sedimentary environments (Fig. 3a,b, Supplementary Tables S1, S2). In core A1, the peat includes only freshwater diatom species whereas the sand layers are dominated by brackish-water and marine diatoms. Common species in the peat include the freshwater species *Aulacoseira crassipunctata*. Dominant species in sand B are the brackish-marine species *Paralia sulcata* and the marine species *Thalassiosira* spp. and *Delphineis surirella*. Sand A contains mixed assemblages including the freshwater species *Aulacoseira crassipunctata* and *Tabellaria fenestrata* and the freshwater-brackish species *Rhopalodia gibba* (Fig. 3). In slice D1, sand C contains mixed assemblages including freshwater and brackish-marine diatoms (*Hantzschia amphioxys*, *Luticola mutica*, *Thalassiosira* spp., and *Delphineis surirella*), whereas the underlying sand D is dominated by the marine diatoms *Delphineis surirella* and *Triceratium* sp. (Fig. 3). The peat layer above sand C includes fruits of the aquatic plant *Potamogedon* (Fig. 3d). Diatom species identified in the peat underlying sand layers A and B (e.g. *Aulacoseira crassipunctata*, *Hantzschia amphioxys*, and *Luticola mutica*) have been reported in non-fluvial freshwater swamp and marsh, maintained by slow peat decomposition depending on low temperature (ca.15 degrees Celsius in summer and ca. − 3 degrees Celsius in winter)^23^ and moderate annual precipitation (ca. 1000 mm)^23^, in eastern Hokkaido^24,25^.

Ages in the peat were estimated by tephrochronology and radiocarbon dating. Energy dispersive X-ray analyses revealed two distinct populations of tephra shards in the cross-plots of K_2_O and TiO_2_ (Fig. 3c). Those with higher and lower K_2_O contents overlap with the Mount Tarumai and Mount Komagatake tephras, respectively. On the basis of their chemical components, historical eruptions, previously reported distributions of local tephras, and shallow stratigraphic position, we assigned the top and middle volcanic ash layers to the Tarumai-a (1739 CE) and Komagatake-c2 (1694 CE) tephras, respectively. The lowest volcanic ash layer is possibly correlated with the Komagatake-d (1640 CE) tephra, although this tephra is very sparsely distributed in eastern Hokkaido^26,27^. The Komagatake-d tephra lies about 10 cm above the base of the peat in slice D1 (Fig. 3a), but radiocarbon dating of the peat suggests that there is a depositional hiatus below the tephra. Two leaf samples from just below the tephra yielded calendar ages of 1522–1950 CE (Supplementary Table S3), whereas plant macrofossils a few centimeters beneath them yielded ages of ~ 1260–1450 CE (Fig. 3a, Supplementary Table S3). In core A1, a radiocarbon age of ~ 1300–1410 CE was obtained from plant macrofossils just below sand B near the base of the peat (Fig. 3b, Supplementary Table S3).

## Discussion

The series of oval ponds is interpreted as scour ponds resulting from coastal erosion that were subsequently isolated from the sea. Typical ponds on a beach ridge plain are parallel to the ridge’s trend defined by inter-ridge swales as a result of shoreline progradation^28,29^ (Fig. 1b). However, the oval ponds at Kiritappu marsh have their long axes perpendicular to the shoreline and are on the seaward edge of the beach ridge. The GPR profiles show an erosional unconformity beneath the ponds. The lithological evidence shows that the unconformity cuts through what was shown by diatom assemblages as an intertidal or subtidal sandy beach. These observations suggest that the ponds were subjected to rapid and energetic erosional scouring, such as extreme waves, rather than surface erosion by blowout^30,31^ due to strong airflow, or natural shoreline progradation.

The hydrological setting of this study site and decadal series of aerial photographs can eliminate fluvial and anthropogenic origins of the oval ponds. The southern part of the Kiritappu marsh is occupied by meandering streams, while northern part is maintained mainly by beach ridges and inter-ridge ponds and marshes (Fig. 1b). Discharging water goes through the southern meandering streams, and the inter-ridge wetlands are replenished only by rainfall and surface seepage. The fluvial activities therefore hardly affect the beach ridge near the oval ponds. Aerial photographs taken by the Geospatial Information Authority of Japan show that the oval ponds have not changed in seven decades, ruling out recent flooding and anthropogenic influences as the origin of the ponds (Supplementary Fig. S4).

The stratigraphic evidence in slice D1 represents changes in environments from erosional breaching, deposition by extreme waves, and isolation of the scour pond. The CT image of slice D1 shows an unconformity between sand C and underlying sand D, plus peaty soil clasts within sand C implying that a high-energy current eroded the underlying soil and incorporated it into the sand. Diatom assemblages in sand C contain both marine species and freshwater species, especially those preferring dry environments, indicating that the sand was derived from both freshwater and coastal environments. These observations are consistent with coastal erosion by extreme waves, followed by the transport and redeposition of sediment from freshwater and coastal environments. The presence of peat including freshwater diatom species above the sand layer shows that the pond at this site was subsequently isolated from the sea.

Written records of historical storms can serve as a guide to the likelihood of breaching the coast by storm waves. These records show that the Kiritappu marsh has not been flooded during storms for the past 200 years. The earliest written records in eastern Hokkaido are chronicles kept by monks of the Kokutaiji Temple in Akkeshi (Fig. 1a), which was built in 1804 CE by the Tokugawa Shogunate. These and more recent archives record 37 storms and flooding events in the Akkeshi and Kiritappu regions^32,33^ (Supplementary Table S4). They mainly describe storm damage to farms and fisheries, due probably to wind and rain, and have no clear descriptions of coastal erosion. It is difficult to entirely rule out unusually large storm erosion events on the millennial time scale as a cause of the scour ponds in Kiritappu, but waves capable of breaching the Kiritappu coast are most likely to be tsunami waves produced by long ruptures in the southern Kuril trench.

According to studies of recent giant tsunamis^12–17^, scour ponds are isolated by drift sand promptly after their formation by rapid recovery of the contemporary shoreline, therefore we can use ages determined from their deposits to constrain the time of their formation. Radiocarbon ages just above sand C suggest that the scour pond was enclosed shortly before 1260–1450 CE at the site of slice D1. This age is consistent with the previously reported chronology of prehistoric outsized tsunamis in eastern Hokkaido^3,4,8,9^. Varved sediments at Harutori-ko, about 60 km west of the study site, show that the most recent unusually large tsunami occurred in the seventeenth century, probably around 1625–1635 CE^34,35^. The modeled rupture area of the seventeenth century tsunami includes both the Tokachi-oki and Nemuro-oki segments of the Kuril trench (green rectangle in Fig. 1a)^3,5,7^. The penultimate tsunami, estimated to have occurred in the thirteenth–fourteenth century^1,3,4,8^, can be correlated with the enclosure of the scour pond at the site of slice D1. The sedimentary hiatus indicated by the radiocarbon ages and the absence of deposits from the seventeenth century tsunami may reflect extensive erosion of peat by the seventeenth century tsunami, as has been documented in a pond after the 2011 Tohoku tsunami^36^.

Sandy deposits in other scour ponds also correspond to tsunami deposits generated by the seventeenth century tsunami and its predecessor. Freshwater peat layers include one or two layers of graded sand (Fig. 3) containing mixed diatom assemblages, which suggest that they were emplaced by high-energy marine incursions in a calm environment, and sedimentary features that include grading of the sand layer and a sharp lower contact with underlying peat are consistent with tsunami deposits^37–39^. The younger of these sand layers underlies the historical Komagatake-c2 ash layer, and plant macrofossils just below the older sand layer yielded a radiocarbon age of ~ 1300 to 1410 CE at the site of core A1. We correlate these sand layers to those identified as tsunami deposits KS3 and KS4 respectively by Nanayama et al.^4^ and K2 and K3 respectively by Sawai et al.^9^ along the Kiritappu coast.

The scour ponds in the Kiritappu marsh probably represent a combination of those newly formed by breaches during the penultimate tsunami event in the thirteenth–fourteenth century, and relict scour ponds in abandoned breaches that hold the thirteenth–fourteenth century tsunami deposits. As observed in recent^1,12–21^ and paleo^22^ tsunamis, tsunami waves not only create new breaches but also erode breaches generated by earlier events. Ages obtained from the base of a peat deposit and the presence of tsunami deposits within a scour pond therefore can constrain the time of its isolation after the most recent erosional event. The evidence from tsunami deposits, tephrochronology, and radiocarbon dating shows that the scour ponds in the study site might be the result of at least two generations of erosion by tsunami waves, one in the thirteenth–fourteenth century (at the site of slice D1) and an earlier generation (at the site of core A1).

The presence of relict scour ponds eroded in different generations may also suggest that the shoreline has not prograded in a simple seaward progression of new beach ridges. Details of sea-level changes after the Holocene highstand have been reconstructed by fossil diatom assemblages within coastal deposits in Akkeshi and Onnetoh (Fig. 1a)^1,40,41^. The assemblages show at least four relative sea-level falls during the last 3000 years^40,41^. Among these, three of the four sea-level falls were attributed to coastal uplift associated with postseismic stress release^41^. The beach ridges of Kiritappu marsh also have been interpreted as the result of the coastal uplift events associated with decades-long postseismic slip along parts of the plate interface deeper than the seismogenic zone after multi-segment earthquakes, such as the seventeenth century earthquake^11,42^. In this scenario, a new foredune appears farther seaward each time a former foredune becomes abandoned above the tidal range during postseismic coastal uplift^42^. If this is true, older scour ponds should tend to occur farther inland. However, scour ponds of different ages occupy the same beach ridge at the study site, probably because the contemporary shoreline has undergone repeated transgression and regression between the beach ridge and the present shoreline, not uniform progradation, between the thirteenth–fourteenth century and the older event. The transgression/regression hypothesis is consistent with cyclic changes of interseismic subsidence and postseismic uplift reconstructed by coastal geology and micropaleontology^1,10,25^. For example, the most recent multi-segment earthquake in the seventeenth century was preceded by rapid subsidence, as quantitatively shown by a diatom-based transfer function for elevation reconstructions^11^. The preseismic subsidence caused coastal transgression and eroded the contemporary shoreline. Such transgressive erosions due to earthquake cycles would tend to interrupt shoreline progradation. A similar episode may have preceded the thirteenth–fourteenth century and earlier tsunamis. The recent rapid subsidence evident from space geodetic and tide gauge data^43,44^ may lead to shoreline erosion around the relict scour ponds in the absence of human disturbances, but today an artificial tidal embankment prevents it.

We studied the scour ponds in Kiritappu marsh to better understand prehistoric tsunami damage on the coast. The well-preserved marsh permitted us to carry out high-resolution photogrammetry to identify trough-shaped depressions, and evidence from GPR and coastal geology helped in their interpretation. This integration of geodetic and geologic techniques enable us to reconstruct coastal erosion during the unusually large tsunamis of the thirteenth–fourteenth century and possibly older times. Our approach can be applied in similar settings elsewhere with beach-ridge plains, helping to reconstruct the full history of tsunamis, the coastal damages they cause, and their effects on the position of the shoreline.

## Methods

### Photogrammetry

Structure-from-motion multi-view stereo photogrammetry was carried out using a quadcopter unmanned aerial vehicle (UAV) with an optical camera and the network Real Time Kinematic Global Navigation Satellite System (network RTK-GNSS) (Phantom 4 RTK, SZ DJI Technology Co., Ltd., Shenzhen, China). The survey was performed in mid May 2021. The ground sample distance (GSD) was 1.27 cm in the mapping project. The vertical overlap of consecutive photographs along the same heading of the UAV in the flight path was 90%. The horizontal overlap, which is based on pairs of photographs on two parallel paths, was 60%.

Photographic data were processed with Pix4D software (Pix4D Inc., Prilly, Switzerland)^45^ to create orthomosaics and georeferenced DSMs. The software automatically merged photographs taken by the UAV and created a point cloud dataset. The DSM produced from the point cloud dataset was then projected using ArcGIS software (ESRI Inc., Redlands, California, USA)^46^.

### Ground-penetrating radar survey

The GPR survey employed the Pulse Ekko system (Sensors & Software Inc., Mississauga, Ontario, Canada)^47^ using high-frequency electromagnetic waves for mapping lithology below the ground surface. The survey used bistatic, shielded 250 MHz antennae with a 165 V transmitter. Transects parallel to the present shoreline crossed a series of ponds and seaward edges of the beach ridge (transects A–D, S4–S9). Transects perpendicular to the shoreline crossed the scarp of the beach ridge (transects S1–S3). The GPR data were acquired in October 2020, February and May 2021, and May 2022. Common midpoint surveys were conducted on swamp, beach ridges, and frozen ice in each season to estimate the velocity of the radar waves. Elevations of the GPR transects were measured using a network RTK-GNSS receiver (Leica Geosystems GS07 with a CS20 controller, Leica Geosystems AG, Heerbrugg, Switzerland).

The GPR data were processed with Reflexw software (Sandmeier Scientific Software, Karlsruhe, Germany). Data processing included dewow filtering, zero-time corrections, bandpass filtering, gain control, time–depth conversion, and static corrections. The radar wave velocity was calculated from common midpoint surveys and used for the time–depth conversion. The calculated velocity was 0.05 m/ns.

### Sediment samples

Sediment samples were collected using a Russian sampler and a geoslicer. To collect the sliced samples from the bottom of the ponds, the geoslice sampler was operated on frozen ice through drilled holes in the winter season of 2020. The collected samples were logged visually in the field and later were imaged by CT to clarify sedimentary structures. The CT images were taken using the Hitachi Supria Grande PREMIUM (Hitachi, Ltd., Tokyo, Japan) at the Geological Survey of Japan.

### Fossil diatom analysis

Twenty-six subsamples were collected to cross stratigraphic changes from core A1 and slice D1 for fossil diatom analysis. The subsamples were prepared by the procedure of Sawai et al.^40^ for identifying and counting diatoms. At least 250 diatom valves were identified under a light microscope at 600 × magnification for each prepared slide. Diatom identification followed standard^48–56^ and local^57,58^ literatures. Results are reported as percentages of all diatom valves counted in each sample. Ecological interpretation of diatom species was based on international^48–56,59^ and local^24,25,57,58^ compilations.

### Identification of volcanic ash layers

Positions of volcanic ash layers were determined by eye in the field and in CT images in the laboratory. The ash samples were ultrasonicated in the laboratory before analysis. The elemental concentrations of 10 glass shards from each sample were measured with an energy dispersive X-ray microanalyzer (EDX) by Furusawa Geological Survey Inc., Okazaki, Japan. Cross-plots of K_2_O and TiO_2_ from the EDX results were used to identify the origin of the ash layers, as established by previous studies^26,60^.

### Radiocarbon dating

A sediment sample was gently washed through 1 mm, 0.5 mm and 0.25 mm mesh sieves. Plant macrofossils (Fig. 3d) in each fraction were picked out under a binocular microscope. Plant macrofossils were dated by accelerator mass spectrometry at Beta Analytic Inc., Miami, USA. Calibration of radiocarbon ages employed the OxCal 4.4^61^ program with the IntCal20 radiocarbon calibration dataset^62^.

## Supplementary Information

Supplementary Information.

## Data Availability

All data integral to the stated conclusions are presented within the paper and the Supplementary Information.
