# Supplementary material for: Scour ponds from unusually large tsunamis on a beach-ridge plain in eastern Hokkaido, Japan
Source: Sci Rep. 2023 Feb 21;13:3064. doi: 10.1038/s41598-023-30061-9 (PMC9944914; doi:10.1038/s41598-023-30061-9)
Supplement: Supplementary file 1 — Supplementary Information. [file 41598_2023_30061_MOESM1_ESM.pdf]

Sawai et al. Supplementary Fig. S1

(a) Aerial photograph showing the northeastern coast of Kiritappu marsh (Geospatial Information Authority of Japan image CHO-78-07-06B-0012). Two lines of natural ponds and swales (arrows) are parallel to the present shoreline. An artificial tidal embankment separates the marsh and the present beach. The portion of this photo shown in Fig. 2a is outlined. (b) Quadcopter image (location in a) showing coastal features and the surface expression of scour ponds.

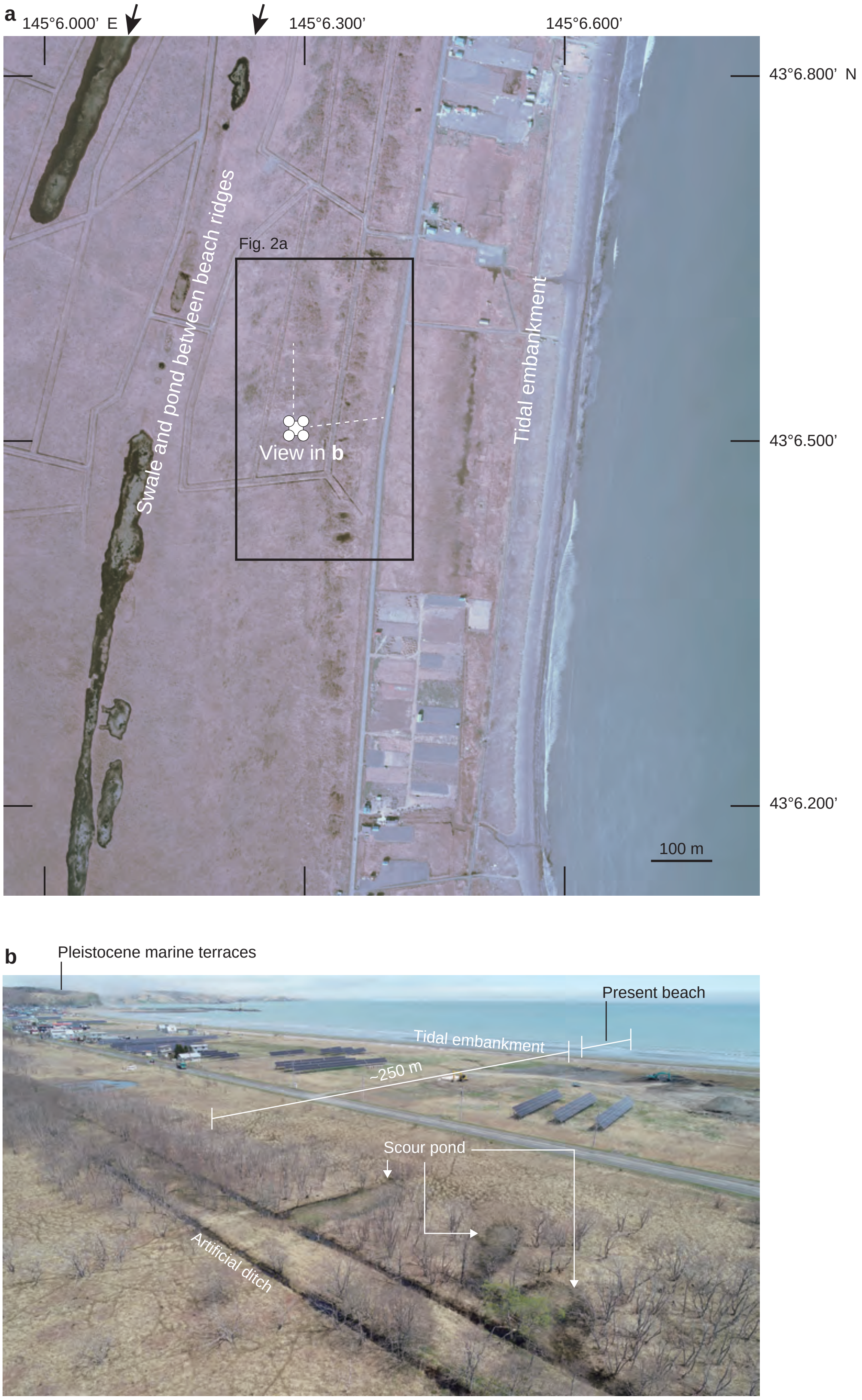

Sawai et al. Supplementary Fig. S2

GPR images along the transects perpendicular to the shoreline (locations in Fig. 2a). Vertical exaggeration is approximately 4x.

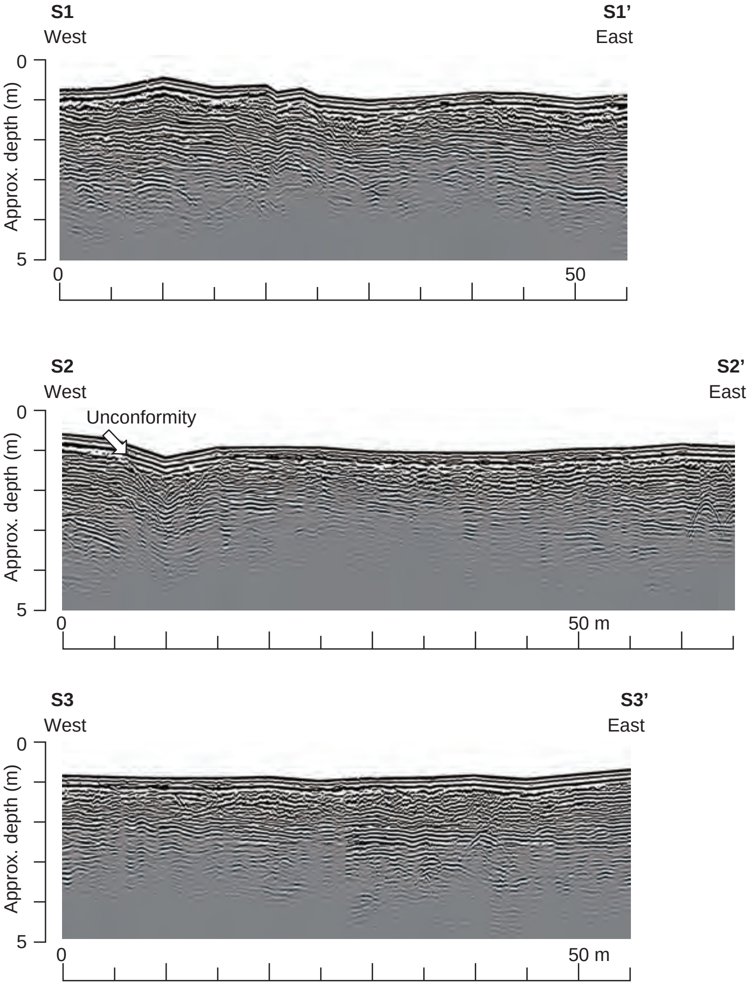

Sawai et al. Supplementary Fig. S3

GPR images along the transects parallel to the shoreline (locations in Fig. 2a). Vertical exaggeration is approximately 4x.

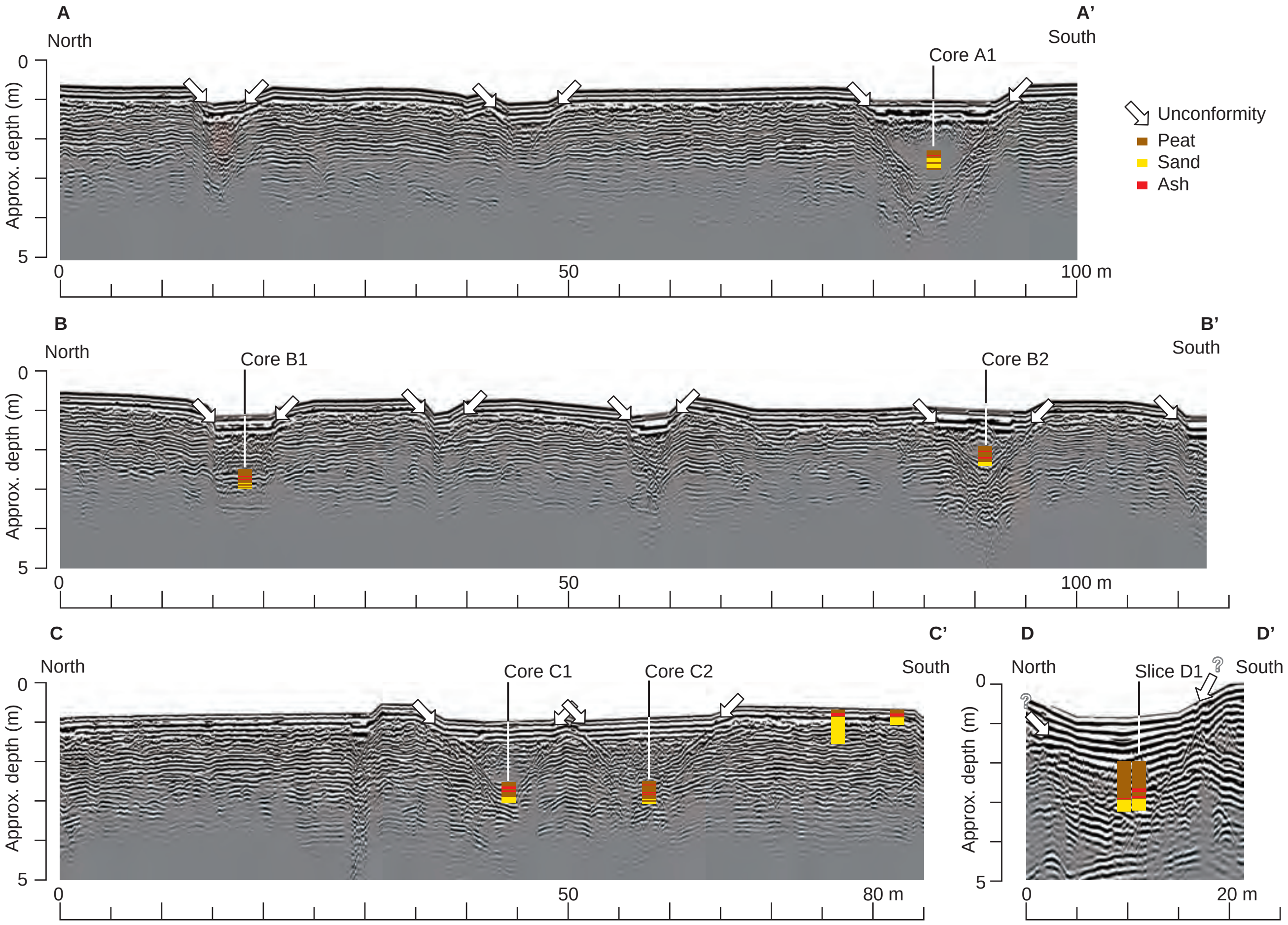

Sawai et al. Supplementary Fig. S3, *continued*

GPR images along the transects parallel to the shoreline.

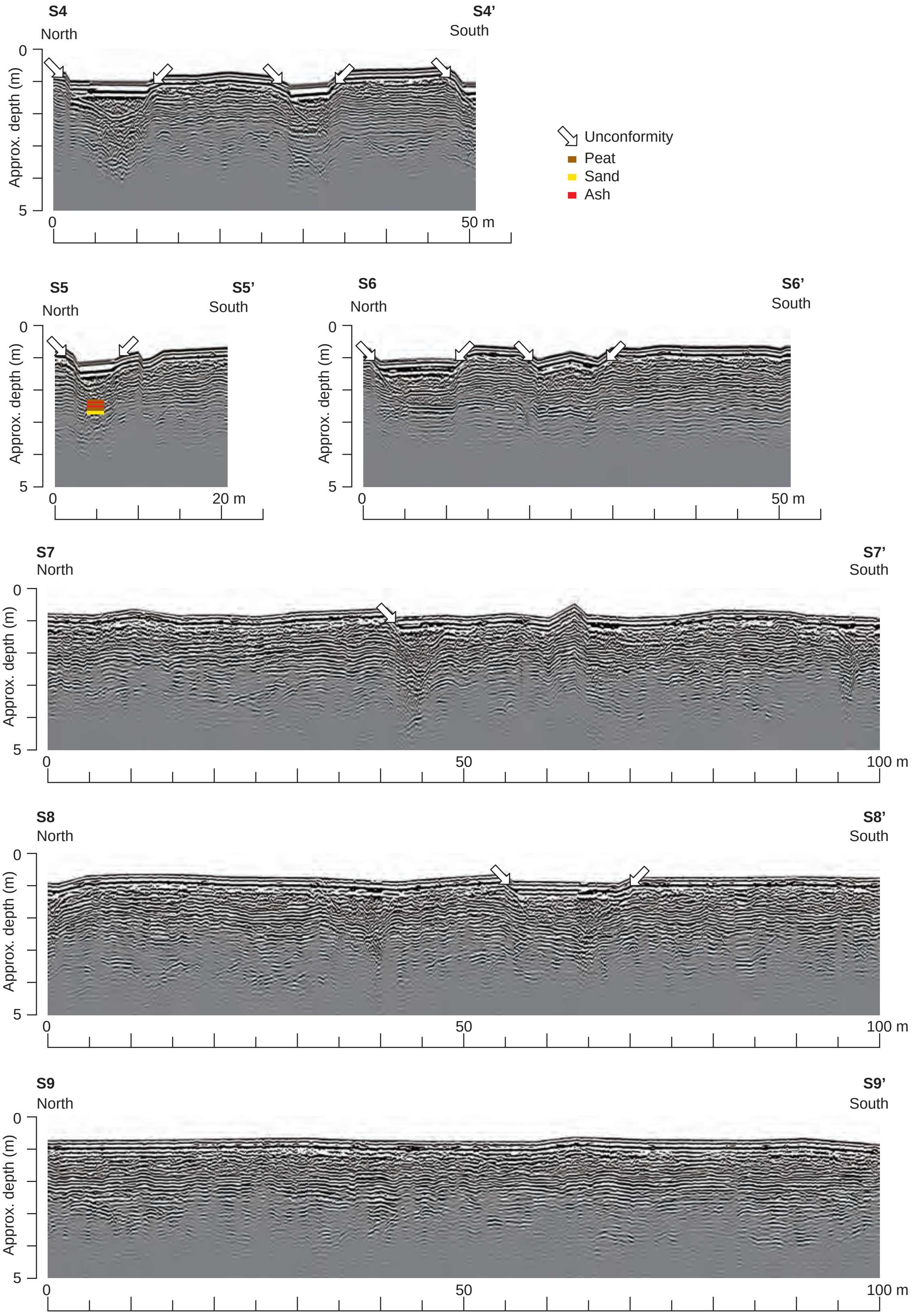

Sawai et al. Supplementary Fig. S4

Historical aerial photographs of the study area. Photographs from Geospatial Information Authority of Japan, images (a) USA-M107-53, (b) CHO-78-07-06B-0012, (c) HO901X-C17-7, and (d) HO20056X-C6-21.

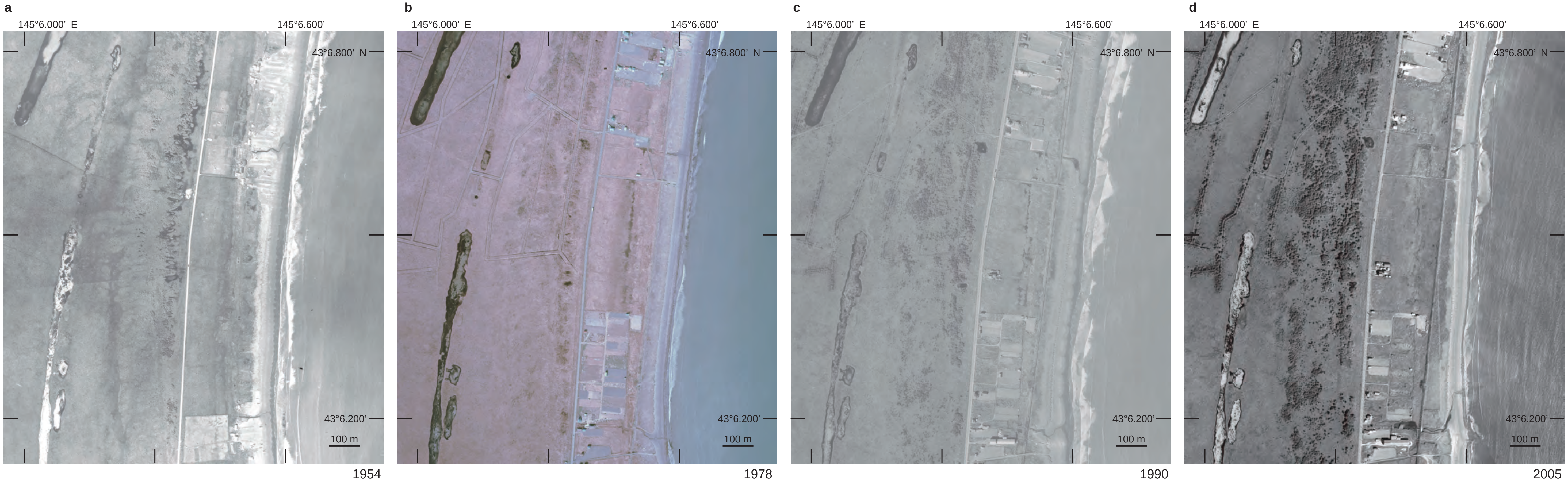

Sawai et al. Supplementary Table S1. Diatom assemblages from slice D1.

**Species name / Depth**	88	91	94	97	100	103	110	115	**Habitat**	**Reference**
Achnanthidium minutissimum (Kützing) Czarnecki	0	0.416667	0	0	0	0	0	0	Freshwater	Kobayasi et al. (2006)
Amphora sp.	0	0	0	0.847458	0	0	0	0	Unknown	
Aulacoseira crassipunctata Krammer	0.440529	4.583333	0	0	0	0	0	0	Freshwater, epiphytic	Krammer & Lange-Bertalot (1991)
Aulacoseira granulata (Ehrenberg) Simonsen	0.440529	0.416667	0	0	0	0.913242	0	0	Freshwater, planktonic	Kobayasi et al. (2006)
Caloneis amphisbaena (Bory) Cleve	0.440529	0.416667	0	0	0	0	0	0	Freshwater, epipelon, epiphytic	Patrick & Reimer (1966)
Chaetoceros resting spores	0	0	1.908397	3.389831	0	1.369863	0	0	Planktonic, Marine	Hendey (1964)
Cocconeis placentula Ehrenberg	0	0	0	0.423729	0	0	0	0	Freshwater, epiphytic	Kobayasi et al. (2006)
Cocconeis placentula var. *euglypta* (Ehrenberg) Cleve	0	0	0	0	0.413223	0	0	0	Freshwater, epiphytic	Kobayasi et al. (2006)
Cocconeis scutellum Ehrenberg	0	0	1.908397	0.847458	0	1.369863	0	0.877193	Brackish, epiphytic	Vos & de Wolf (1988)
Cyclotella atomus var. *gracilis* Genkal & Kiss	0	0	0	0	0	0	0	0.877193	Freshwater-brackish	Kobayasi et al. (2006)
Cyclotella meneghiniana Kützing	0	0	0	0	0	0	1.960784	0	Brackish-marine, planktonic	Vos & de Wolf (1988)
Cymbella cistula (Ehrenberg) O.Kirchner	0	0.416667	0	0	0	0	0	0	Freshwater	Patrick & Reimer (1975)
Cymbella cuspidata W.Smith	0	0.416667	0	0	0	0	0	0	Freshwater	Krammer & Lange-Bertalot (1986), Patrick & Reimer (1966)
Cymbella proxima Reimer	0	0.833333	0	0	0	0	0	0	Freshwater	Patrick & Reimer (1975)
Cymbella turgidula Grunow	0	0.416667	0	0	0	0	0	0	Freshwater	Patrick & Reimer (1975)
Cymbopleura naviculiformis (Auerswald) Krammer	0.440529	0.416667	0	0	0	0	0	0	Freshwater	Patrick & Reimer (1975)
Delphineis sp.	0	0	0	1.271186	0	0	0	0	Unknown	
Delphineis surirella (Ehrenberg) G.W.Andrews	0	0	11.83206	50.42373	1.239669	26.94064	0	18.42105	Brackish-marine	Hendey (1964), Witkowski et al. (2000)
Encyonema gracile Krammer	0.440529	0	0	0	0	0	0	0	Freshwater	Krammer & Lange-Bertalot (1986), Patrick & Reimer (1966)
Encyonema minutum (Hilse) D.G.Mann	0.440529	3.75	0	0	0	0	0	0	Freshwater	Krammer & Lange-Bertalot (1986), Patrick & Reimer (1966)
Encyonema silesiacum (Bleisch) D.G.Mann	2.202643	0.416667	0	0	0	0	0	0	Freshwater	Krammer & Lange-Bertalot (1986), Patrick & Reimer (1966)
Epithemia adnata (Kützing) Brébisson	0.440529	0	0	0	0	0	0	0	Freshwater, epiphytic	Patrick & Reimer (1975)
Epithemia sp.	0.440529	0	0	0	0	0	0	0	Freshwater	Krammer & Lange-Bertalot (1991)
Eunotia minor (Kützing) Grunow	2.643172	0	0	0	0	0.456621	0	0	Freshwater	Krammer & Lange-Bertalot (1991)
Eunotia pectinalis (Kützing) Rabenhorst	0.881057	0	0	0	0	0	0	0	Freshwater	Krammer & Lange-Bertalot (1991)
Eunotia praerupta Ehrenberg	0.440529	0	0	0	0	0	0	0	Freshwater	Krammer & Lange-Bertalot (1991)
Eunotia veneris (Kützing) De Toni	1.321586	0	0	0	0	0	0	0	Freshwater	Krammer & Lange-Bertalot (1991)
Fragilaria ? sp.	0	0	3.816794	0	0	0	0	0	Unknown	
Fragilariaceae spp.	22.02643	21.25	2.290076	0	2.892562	0	0	0	Unknown	
Gomphonema acuminatum Ehrenberg	0.440529	0	0	0	0	0	0	0	Freshwater	Krammer & Lange-Bertalot (1986), Patrick & Reimer (1975)
Gomphonema augur Ehrenberg	0	1.666667	0	0	0	0	0	0	Freshwater	Krammer & Lange-Bertalot (1986), Patrick & Reimer (1975)
Gomphonema gracile Ehrenberg	0	0	0	0	0.413223	0	0	0	Freshwater	Krammer & Lange-Bertalot (1986), Patrick & Reimer (1975)
Gomphonema parvulum (Kützing) Kützing	0.881057	0.416667	0	0	0	0	0	0	Freshwater	Krammer & Lange-Bertalot (1986), Patrick & Reimer (1975)
Gomphonema pseudoaugur Lange-Bertalot	0	0.416667	0	0	0	0	0	0	Freshwater	Krammer & Lange-Bertalot (1986)
Gomphonema truncatum Ehrenberg	0	1.666667	0	0	0	0	0	0	Freshwater	Krammer & Lange-Bertalot (1986), Patrick & Reimer (1975)
Grammatophora marina (Lyngbye) Kützing	0	0	0.381679	0	0	0	0	0	Marine	Hendey (1964), Witkowski et al. (2000)
Hantzschia amphioxys (Ehrenberg) Grunow	0	0	0	0.847458	16.94215	0	0	0	Freshwater, subaerial	Krammer & Lange-Bertalot (1986)
Humidophila contenta (Grunow) R.L.Lowe & al.	0	0	0	0	5.371901	0	0	0	Freshwater, subaerial	Krammer & Lange-Bertalot (1986)
Luticola mutica (Kützing) D.G.Mann	0	0	0	0	12.80992	0	0	0	Freshwater-brackish, subaerial	Patrick & Reimer (1966)
Mastogloia smithii Thwaites ex W.Smith	0	0	0.381679	0	0	0	0	0	Brackish	Witkowski et al. (2000)
Melosira nummuloides C.Agardh	0	0	2.671756	0	0	0	0	0	Brackish-marine	Witkowski et al. (2000)
Melosira varians C.Agardh	0	0	0.763359	0	0	0	0	0	Freshwater	Kobayasi et al. (2006)
Navicula cancellata Donkin	0	0	0	0.847458	0	0.913242	0	0	Brackish-marine	Hendey (1964)
Navicula cryptotenella Lange-Bertalot	0	0	0	0.423729	0	0	0	0	Brackish	Krammer & Lange-Bertalot (1986)
Navicula gregaria Donkin	0	0	0.381679	0	0	0	0	0	Brackish	Sawai et al. (2016)
Navicula libonensis Schoeman	0	0.833333	1.526718	0.423729	0	0	0	0	Freshwater-brackish	Witkowski et al. (2000)
Navicula peregrina (Ehrenberg) Kützing	0	1.25	0.763359	0	0	0	0	0	Brackish-marine	Vos & de Wolf (1988)
Navicula peregrinopsis Lange-Bertalot & Witkowski	0	0	0.763359	0	0	0	0	0	Brackish-marine?	Witkowski et al. (2000)
Navicula radiosa Kützing	7.929515	8.333333	0	0	0	0	0	0	Freshwater	Patrick & Reimer (1966)
Navicula rhynchocephala Kützing	0	0.416667	9.923664	0.847458	0	0	0	0	Brackish-marine	Vos & de Wolf (1988)
Navicula salinarum Grunow	0	0	3.435115	0	0	0	0	0	Brackish-marine	Vos & de Wolf (1988)
Navicula slesvicensis Grunow	0	0.833333	0	0	0	0	0	0	Brackish	Sawai et al. (2016)
Navicula sp.1	0.440529	0	0	0	0	0	0	0	Unknown	
Navicula sp.2	0	0	0.763359	0	0	0	0	0	Unknown	
Navicula (?) sp.3	0	0	0	0.423729	0	0	0	0	Unknown	
Neidium bisulcatum (Lagerstedt) Cleve	0.440529	1.666667	0	0	0	0	0	0	Freshwater	Patrick & Reimer (1966)
Neidium iridis (Ehrenberg) Cleve	0.881057	1.25	0	0	0	0	0	0	Freshwater	Patrick & Reimer (1966)
Nitzschia brevissima Grunow	0	0	0	0	0.413223	0	0	0	Freshwater-brackish, subaerial	Krammer & Lange-Bertalot (1988), Sawai et al. (2016)
Nitzschia pura Hustedt	0	0	0.763359	0	0	0	0	0	Brackish	Krammer & Lange-Bertalot (1988), Sawai et al. (2016)
Odontella aurita (Lyngbye) C.Agardh	0	0	0	2.542373	0	0	0	0	Marine, planktonic	Vos & de Wolf (1988)
Paralia sulcata (Ehrenberg) Cleve	0	0	25.57252	8.898305	20.66116	28.76712	0	9.649123	Marine, planktonic	Vos & de Wolf (1988)
Pinnularia borealis Ehrenberg	0	0	0	0	7.024793	0	0	0	Freshwater, epipelic, subaerial	Vos & de Wolf (1988)
Pinnularia divergens W.Smith	0	0	0	0	0.413223	0	0	0	Freshwater	Patrick & Reimer (1966)
Pinnularia interrupta W.Smith	0.440529	0	0	0	0	0.456621	0	0	Freshwater	Patrick & Reimer (1966)
Pinnularia lagerstedtii (Cleve) A.Cleve	0	0	0	0	2.479339	0	0	0	Freshwater-brackish, aerophilic	Krammer & Lange-Bertalot (1986), Krammer (2000)
Pinnularia notabilis Krammer	0	0	0	0	1.239669	0.456621	0	0	Freshwater	Krammer (2000)
Pinnularia viridis (Nitzsch) Ehrenberg	0.440529	0	0	0	0	0	0	0	Freshwater	Krammer (2000)
Pinnunavis yarrensis (Grunow) H.Okuno	0	0	0	0	0.413223	0	0	0	Brackish	Sawai & Nagumo (2003)
Planothidium delicatulum (Kützing) Round & Bukhtiyarova	0	0	0.763359	0	0	0	0	0	Brackish-Marine, Epipsammic	Vos & de Wolf (1988)
Pseudostaurosira brevistriata (Grunow) D.M.Williams & Round	0	0	0	0	0.413223	0.456621	0	0	Freshwater	Patrick & Reimer (1966)
Rhaphoneis amphiceros (Ehrenberg) Ehrenberg	0	0	0	0	0	2.283105	0	7.017544	Brackish-marine	Hendey (1964), Witkowski et al. (2000)
Rhopalodia gibba (Ehrenberg) O.Müller	1.321586	4.166667	0	0	0	0	0	0	Freshwater-brackish, epiphytic	Vos & de Wolf (1988)
Sellaphora pupula (Kützing) Mereschkovsky	2.643172	0.833333	0	0	0	0	0	0	Freshwater	Patrick & Reimer (1966)
Sellaphora rectangularis (W.Gregory) Lange-Bertalot & Metzeltin	0.440529	1.25	0	0	0	0	0	0	Freshwater	Patrick & Reimer (1966)
Stauroneis phoenicenteron (Nitzsch) Ehrenberg	0	0.833333	0	0	0	0	0	0	Freshwater	Patrick & Reimer (1966)
Staurosira neoproducta (Lange-Bertalot) Chudaev & Gololobova	0	2.083333	0	0	0	0	0	0	Freshwater	Krammer & Lange-Bertalot (1991)
Staurosira venter (Ehrenberg) Cleve & J.D.Möller	0	1.25	0	0	0	0	0	0.877193	Freshwater	Patrick & Reimer (1966)
Stephanodiscus sp.	0	0	0	0	0	0.456621	0	0	Freshwater, planktonic	
Surirella marina Krammer	0	0	0.381679	0	0	0	0	0	Brackish	Sawai et al. (2016)
Tabellaria fenestrata (Lyngbye) Kützing	42.29075	29.58333	2.290076	0	0.413223	0	0	0	Freshwater	Patrick & Reimer (1966)
Tabellaria flocculosa (Roth) Kützing	0.440529	0.833333	0	0	0	0	0	0	Freshwater	Patrick & Reimer (1966)
Thalassionema nitzschioides (Grunow) Mereschkowsky	0	0	1.526718	1.271186	4.132231	0	0	0	Marine, planktonic	Hendey (1964)
Thalassiosira spp.	0	0	22.1374	19.49153	15.28926	30.13699	0	0	Marine, planktonic	
Trachyneis aspera (Ehrenberg) Cleve	0	0	0	0	0	0	0	1.754386	Brackish-marine	Hendey (1964)
Triceratium sp.	0	0	0	0	0	0	94.11765	58.77193	Marine	Round et al. (1990)
Tryblionella salinarum (Grunow) Pelletan	0	0	0	0	0.413223	0	0	0	Brackish	Witkowski et al. (2000)
Ulnaria ulna (Nitzsch) Compère	0.440529	0	0	0	0	0	0	0	Freshwater	Patrick & Reimer (1966)
Ulnaria sp.	2.202643	0	0.763359	0	0	0	0	0	Freshwater	
Unknown	5.286344	6.666667	2.290076	6.779661	6.61157	5.022831	3.921569	1.754386		
**Total diatoms counted**	227	240	262	236	242	219	51	114		

Sawai et al. Supplementary Table S2. Diatom assemblages from core A1.

**Species name / Depth**	140	141	144	147	151	153	154	157	160	162	167	168	**Habitat**	**Reference**
Achnanthidium minutissimum (Kützing) Czarnecki	0	0	0	1.277955	0	4.11985	1.503759	0	0	0	0	0	Freshwater	Kobayasi et al. (2006)
Actinoptychus senarius (Ehrenberg) Ehrenberg	0	0	0	0	0	0	0	0	0	2.764977	0	0	Brackish-marine, planktonic	Hendey (1964)
Amphora sp.	0	0	0	0	0	0	0	0	1.360544	0	0	0	Unknown	
Aulacoseira ambigua (Grunow) Simonsen	3.292181	0	0.392157	0	0	0	0	0	0.680272	0	0	0	Freshwater, planktonic	Kobayasi et al. (2006)
Aulacoseira crassipunctata Krammer	15.63786	12.5	14.11765	11.5016	31.27273	38.57678	16.16541	1.99005	0.680272	0	0.75188	0	Freshwater, epiphytic	Krammer & Lange-Bertalot (1991)
Aulacoseira granulata (Ehrenberg) Simonsen	16.46091	3.225806	2.745098	0.958466	1.090909	0	0	0	0	0	0	0	Freshwater, planktonic	Kobayasi et al. (2006)
Aulacoseira sp.	2.880658	0	0.784314	0	0	0	0	0	1.360544	0	0	0	Freshwater	
Cocconeis scutellum Ehrenberg	0	0	0	0	0	0	0	1.492537	0.680272	1.843318	0	0	Brackish, epiphytic	Vos & de Wolf (1988)
Cyclotella meneghiniana Kützing	0	0	0	0	0	0	0	0	0.680272	0	0	0	Brackish-marine, planktonic	Vos & de Wolf (1988)
Cymbella cistula (Ehrenberg) O.Kirchner	0	0	0	0	0	0.374532	0	0	0	0	0	0	Freshwater	Patrick & Reimer (1975)
Cymbella cuspidata W.Smith	0	0	0	0.638978	0	0	0	0	0	0	0	0	Freshwater	Krammer & Lange-Bertalot (1986), Patrick & Reimer (1966)
Cymbella mexicana (Ehrenberg) Cleve	0	0	0	0	0	0.374532	0	0	0	0	0	0	Freshwater	Patrick & Reimer (1975)
Cymbella proxima Reimer	0	0	0	0	0	2.996255	0.37594	0	0	0	0	0	Freshwater	Patrick & Reimer (1975)
Cymbella tumida (Brébisson) Van Heurck	0	0	0	0.319489	0.363636	0	0	0	0	0	0	0	Freshwater	Patrick & Reimer (1975)
Cymbopleura naviculiformis (Auerswald) Krammer	0	0	0	0	0.727273	0.749064	0	0	0	0	0	0	Freshwater	Patrick & Reimer (1975)
Decussiphycus hexagonus (Torka) Guiry & Gandhi	0	0	0	0	0.363636	0	0	0	0	0	0	0	Freshwater	Krammer & Lange-Bertalot (1986)
Delphineis surirella (Ehrenberg) G.W.Andrews	0	0	0	0	0	0	0	1.99005	28.57143	39.63134	0.75188	0	Brackish-marine	Hendey (1964), Witkowski et al. (2000)
Denticula ? sp.	0	0	0	0	0	0	0	0	0.680272	0	0	0	Unknown	
Diploneis interrupta (Kützing) Cleve	0	0	0	0	0	0	0	0	0	0.460829	0	0	Brackish-marine	Vos & de Wolf (1988)
Encyonema minutum (Hilse) D.G.Mann	4.938272	0.806452	1.176471	0.638978	0.363636	0	1.879699	0.995025	2.721088	0	0	0	Freshwater	Krammer & Lange-Bertalot (1986), Patrick & Reimer (1966)
Encyonema silesiacum (Bleisch) D.G.Mann	1.234568	2.419355	3.137255	0.319489	0	0	0	0.995025	0.680272	0	0	0	Freshwater	Krammer & Lange-Bertalot (1986), Patrick & Reimer (1966)
Entomoneis alata (Ehrenberg) Ehrenberg	0	0	0	0	0	0	0	6.467662	0	0	0	0	Brackish	Witkowski et al. (2000)
Eunotia minor (Kützing) Grunow	0	1.612903	0	0.958466	1.090909	0	0	0	0	0	3.383459	0.408163	Freshwater	Krammer & Lange-Bertalot (1991)
Eunotia pectinalis (Kützing) Rabenhorst	0	2.016129	1.568627	1.277955	1.818182	0	0	0	0	1.382488	0.75188	0	Freshwater	Krammer & Lange-Bertalot (1991)
Eunotia praerupta Ehrenberg	0	0	0	0	0.363636	0.749064	0	0	0	0	1.12782	0	Freshwater	Krammer & Lange-Bertalot (1991)
Eunotia spp.	0	0	0.784314	0	0	0	0	0.995025	0	0	0.75188	0	Freshwater	
Fragilaria sp.	0	0	0.784314	0	0	0	6.015038	0	0	0	0	0	Unknown	
Fragilariaceae spp. girdle view	31.68724	24.59677	44.70588	39.9361	21.81818	0	0	1.492537	7.482993	0	0	0	Unknown	
Frustulia rhomboides (Ehrenberg) De Toni	0	7.258065	0	0	0	0	0	0	0	0	0	0	Freshwater	Krammer & Lange-Bertalot (1986)
Frustulia vulgaris (Thwaites) De Toni	0	0	0	1.277955	0	0	0	0	0	0	0	0	Freshwater-brackish	Sawai et al. (2016)
Gomphonema acuminatum Ehrenberg	0	0.806452	0	0.319489	0	0.749064	0	0	0	0	0	0	Freshwater	Krammer & Lange-Bertalot (1986), Patrick & Reimer (1975)
Gomphonema augur Ehrenberg	0	2.016129	0	0.319489	1.090909	0.374532	0	0	0	0	0	0	Freshwater	Krammer & Lange-Bertalot (1986), Patrick & Reimer (1975)
Gomphonema parvulum (Kützing) Kützing	4.938272	0.806452	0.392157	0	0.363636	0	0	0	1.360544	0	0	0	Freshwater	Krammer & Lange-Bertalot (1986), Patrick & Reimer (1975)
Gomphonema sphareophorum Ehrenberg	0	0	0.784314	0	0	0	0	0	0	0	0	0	Freshwater	Krammer & Lange-Bertalot (1986)
Hantzschia amphioxys (Ehrenberg) Grunow	0.411523	0	0	0	0	0	0	0	0	0.460829	21.80451	40	Freshwater, subaerial	Krammer & Lange-Bertalot (1986)
Humidophila contenta (Grunow) R.L.Lowe & al.	0	0	0	0	0	0	0	0	0	0.460829	8.270677	4.489796	Freshwater, subaerial	Krammer & Lange-Bertalot (1986)
Luticola mutica (Kützing) D.G.Mann	0	0	0	0	0	0	0	0	0	0	0.75188	9.387755	Freshwater-brackish, subaerial	Patrick & Reimer (1966)
Navicula digitoradiata (W.Gregory) Ralfs	0	0	0	0	0.363636	0	0	0	0	0	0	0	Brackish-marine	Hendey (1964)
Navicula lapidosa Krasske	0	0	0.392157	0	0	0	0	0	0	0	0	0	Freshwater	Krammer & Lange-Bertalot (1986)
Navicula peregrina (Ehrenberg) Kützing	0	0	0	0	0	0.374532	0.37594	0	0	0	0	0	Brackish-marine	Vos & de Wolf (1988)
Navicula radiosa Kützing	0	1.209677	8.235294	0.958466	4.727273	6.741573	6.766917	0	0	0	0	0	Freshwater	Patrick & Reimer (1966)
Navicula salinarum Grunow	0	0	0	0	1.090909	0	0	0.497512	4.081633	0	0	0	Brackish-marine	Vos & de Wolf (1988)
Navicula slesvicensis Grunow	0	0	0	0	1.454545	0	0	0	0	0	0	0	Brackish	Sawai et al. (2016)
Navicula sp.1	1.234568	0.806452	0	0	0	0	0	0	0	0	0	0	Unknown	
Navicula sp.2	0	0	2.352941	0	0	0	0	0	0	0	0	0	Unknown	
Neidium bisulcatum (Lagerstedt) Cleve	0	0	0	0	0	0	0	0	2.040816	0	0	0	Freshwater	Patrick & Reimer (1966)
Neidium iridis (Ehrenberg) Cleve	0	0.403226	0	2.875399	0	0	0	0	0	0	0	0	Freshwater	Patrick & Reimer (1966)
Nitzschia pura Hustedt	0	0	0	0	0	0	0	38.80597	4.081633	0	0	0	Brackish	Krammer & Lange-Bertalot (1988), Sawai et al. (2016)
Nitzschia scalpelliformis Grunow	0	0	0	0	0	0	0	4.975124	0	0	0	0	Brackish	Witkowski et al. (2000)
Nitzschia sp.1	0	0	0	0	0	0	0	12.93532	1.360544	0	0	0	Unknown	
Nitzschia sp.2	0	0	0	0	0	0	0.37594	8.955224	2.040816	0	0	0	Unknown	
Nitzschia sp.3	0	0	0	0	0	0	0	5.472637	0	0	0	0.408163	Unknown	
Odontella aurita (Lyngbye) C.Agardh	0	0	0	0	0	0	0	0	1.360544	0	0	0	Marine, planktonic	Vos & de Wolf (1988)
Paralia sulcata (Ehrenberg) Cleve	0	0	0	0	0	0	0	0	8.843537	17.51152	0	0	Marine, planktonic	Vos & de Wolf (1988)
Pinnularia borealis Ehrenberg	0	0	0	0	0	0	0	0	0	0.921659	56.76692	42.04082	Freshwater, epipelic, subaerial	Vos & de Wolf (1988)
Pinnularia brevicostata var. *sumatrana* Hustedt	0	0.806452	0	0	0	0	0	0	0	0	0	0	Freshwater	Kawashima & Mayama (2000)
Pinnularia divergens W.Smith	0	0	0	0	0	0	0	0	0	0	0	0.816327	Freshwater	Patrick & Reimer (1966)
Pinnularia interrupta W.Smith	0	2.419355	0	0	0	0	0	0	0	0	0	0	Freshwater	Patrick & Reimer (1966)
Pinnularia lagerstedtii (Cleve) A.Cleve	0	0	0	0	0	0	0	0.497512	0	0	0	0	Freshwater-brackish, aerophilic	Krammer & Lange-Bertalot (1986), Krammer (2000)
Pinnularia nodosa (Ehrenberg) W.Smith	0	0	0	0	0	0	0	0	0	0.460829	0	0	Freshwater	Krammer & Lange-Bertalot (1986),
Pinnularia subgibba Krammer	0	0.403226	0	0	0	0	0	0	0	0	0	0	Freshwater	Krammer (2000)
Pinnularia viridis (Nitzsch) Ehrenberg	0.411523	0.403226	0	0	0	0	0	0	0	0	0	0	Freshwater	Krammer (2000)
Pinnularia sp.1	0	0	0	0	0	0.374532	0	0	0.680272	0	0.75188	0	Freshwater	
Pinnularia sp.2	0.411523	0.403226	0	0	0.363636	0.374532	0	0	0	0	0	0	Freshwater	
Pinnunavis yarrensis (Grunow) H.Okuno	0	0	0	0	0	0	0	0.497512	0	0.921659	0	0	Brackish	Sawai & Nagumo (2003)
Planothidium delicatulum (Kützing) Round & Bukhtiyarova	0	0	0	0	0	0	0	0	0	0	0	0	Brackish-Marine, Epipsammic	Vos & de Wolf (1988)
Rhaphoneis amphiceros (Ehrenberg) Ehrenberg	0	0	0	0	0	0	0	0	0	0.921659	0	0	Brackish-marine	Hendey (1964), Witkowski et al. (2000)
Rhopalodia gibba (Ehrenberg) O.Müller	1.234568	0.806452	0	3.833866	13.09091	13.85768	14.66165	0	0.680272	0	0	0	Freshwater-brackish, epiphytic	Vos & de Wolf (1988)
Sellaphora pupula (Kützing) Mereschkovsky	0.411523	0.403226	1.176471	4.153355	1.454545	1.872659	1.503759	0	0	0	0	0	Freshwater	Patrick & Reimer (1966)
Sellaphora rectangularis (W.Gregory) Lange-Bertalot & Metz	1.234568	2.016129	3.137255	1.916933	0.363636	0.749064	0.37594	0	0	0	0	0	Freshwater	Patrick & Reimer (1966)
Stauroneis phoenicenteron (Nitzsch) Ehrenberg	0	4.435484	0.784314	4.153355	7.636364	0	2.255639	0	0	0	0	0	Freshwater	Patrick & Reimer (1966)
Stauroneis sp.	0	0	0	0	0	1.498127	0	0	0	0	0	0	Freshwater	
Tabellaria fenestrata (Lyngbye) Kützing	6.995885	17.33871	4.705882	8.945687	2.909091	10.48689	39.09774	0	1.360544	0	0	0	Freshwater	Patrick & Reimer (1966)
Tabellaria flocculosa (Roth) Kützing	0.823045	2.822581	1.176471	5.750799	0	6.741573	0	0	0	0	0	0	Freshwater	Patrick & Reimer (1966)
Tabularia fasciculata (C.Agardh) D.M.Williams & Round	0	0	0	0	0	0	0	0	2.721088	0.460829	0	0	Brackish-marine	Vos & de Wolf (1988)
Thalassionema nitzschioides (Grunow) Mereschkowsky	0	0	0	0	0	0	0	0	0.680272	0.921659	0	0	Marine, planktonic	Hendey (1964)
Thalassiosira (marine) spp.	0	0	0	0	0	0	0	1.99005	14.28571	27.18894	0	0	Marine	
Tryblionella scalaris (Ehrenberg) Siver & P.B.Hamilton	0	0	0	0.319489	0	0.749064	0.37594	0	0	0	0	0	Brackish	Witkowski et al. (2000)
Tryblionella sp.	0	0	0	0	0	0	0.37594	0	0	0	0	0	Unknown	
Ulnaria ulna (Nitzsch) Compère	0	0.806452	0	0	0	1.123596	0	0	0	0	0	0	Freshwater	Patrick & Reimer (1966)
Ulnaria sp.	0	0	0.392157	0.638978	0	0	0	0	0	0	0	0	Freshwater	
Unknown	5.761317	6.451613	6.27451	6.709265	5.818182	5.992509	7.894737	8.955224	8.843537	3.686636	4.135338	2.44898		
**Total diatoms counted**	243	248	255	313	275	267	266	201	147	217	266	245		

Sawai et al. Supplementary Table S3. Results of radiocarbon dating.

Number in Fig.3	Depth below ground surface/water level	Material	Position	Age (^{14}C BP)	Age (cal BP, CE)	Lab number (Beta-)
1	187-189	Leaves	Below the lower volcanic ash	250±30	429-0, 1522-1950	645152
2	188-189	Leaves	Below the lower volcanic ash	250±30	429-0, 1522-1950	647457
3	190-193	Fruits of *Potamogeton*	Above sand C	710±30	688-564, 1262-1387	645153
4	192-194	Fruits of *Potamogeton*	Above sand C	490±30	546-498, 1404-1452	647458
5	165-168	Seeds	Below sand B	600±30	650-542, 1301-1408	633364

Sawai et al. Supplementary Table S4. Historical and recent storms around the study area.

Date (Gregorian calendar)	Date (Japanese calendar)	Description	Reference
12 September 1879	Meiji 12th year, 9th month, 12th day	Storm. Inundated about 1.5 m (not sure which it is inundation height or distance) at Wangetsu, Akkeshi Town.	Akkeshi Town (1975)
Janunary 1899	Meiji 32nd year, 1st month	Strong wind and snow. Electric poles were collapsed in Akkeshi and Kiritappu Towns.	Akkeshi Town (1975)
31 January 1970	Showa 45th year, 1st month, 31st day	Low pressure. Damage to houses and fishery.	Hamanaka Town (2015)
27 Feburary 1971	Showa 46th year, 2nd month, 27th day	Low pressure. Damage to fishery.	Hamanaka Town (2015)
13 September 1971	Showa 46th year, 9th month, 13th day	Typhoon. Severe damage to fishery.	Hamanaka Town (2015)
14 January 1972	Showa 47th year, 1st month, 14th day	Low pressure. Damage to fishery.	Hamanaka Town (2015)
19 October 1979	Showa 51st year, 10th month, 19th day	Heavy rain by a typhoon. Damage to farming and fishery.	Hamanaka Town (2015)
19 November 1982	Showa 57th year, 11th month, 19th day	Large damage by heavy rain, wind, waves.	Hamanaka Town (2015)
17 March 1983	Showa 58th year, 3rd month, 17th day	Low pressure. Damage to fishery.	Hamanaka Town (2015)
19 July 1984	Showa 59th year, 7th month, 19th day	Heavy rain. Inundation below floor level. Landslide.	Hamanaka Town (2015)
29 August 1986	Showa 61st year, 8th month, 29th day	Heavy rain. Inundation below floor level around Sakaki near our study site.	Hamanaka Town (2015)
24-25 November 1988	Showa 63rd year, 11th month, 24th-25th days	Damage by heavy rain and strong wind to houses, farming, forestry, road, and fishery.	Hamanaka Town (2015)
16-17 August 1989	Heisei 1st year, 8th month, 16th-17th days	Damage by heavy rain and strong wind of a typhoon to agricultural and fisheries facilities.	Hamanaka Town (2015)
5-6 November 1990	Heisei 2nd year, 11th month, 5th-6th days	Low pressure. Damage to fishery and farming.	Hamanaka Town (2015)
10 November 1990	Heisei 2nd year, 11th month, 10th day	Low pressure. Damage to fishery.	Hamanaka Town (2015)
17 Feburary 1991	Heisei 3rd year, 2nd month, 17th day	Low pressure. Damage to houses.	Hamanaka Town (2015)
28 September 1991	Heisei 3rd year, 9th month, 28th day	Damage by a typhoon to agricultural facility.	Hamanaka Town (2015)
11 September 1992	Heisei 4th year, 9th month, 11th day	Typhoon. Inundation below floor level. Damage to fishery.	Hamanaka Town (2015)
19 September 1994	Heisei 6th year, 9th month, 19th day	Damage by a typhoon to fishing vessels.	Hamanaka Town (2015)
16-17 September 1998	Heisei 10th year, 9th month, 16th-17th days	Damage by a typhoon to roads.	Hamanaka Town (2015)
25 September 1999	Heisei 11th year, 9th month, 25th day	Damage by a typhoon to farming, fishery, and houses.	Hamanaka Town (2015)
11-12 September 2001	Heisei 13th year, 9th month, 11th-12th days	Damage by a typhoon to roads and houses.	Hamanaka Town (2015)
1-2 October 2002	Heisei 14th year, 10th month, 1st-2nd days	Damage by a typhoon to houses and warehouses. Inundation below floor level.	Hamanaka Town (2015)
21 June 2003	Heisei 15th year, 6th month, 21st day	Low pressure. Heavy rain. Inundation below and above floor level. Damage to roads.	Hamanaka Town (2015)
23 Feburay 2004	Heisei 16th year, 2nd month, 23rd day	Strong wind and snow. Damage to a cow shed.	Hamanaka Town (2015)
31 August 2004	Heisei 16th year, 8th month, 31st day	Strong wind and waves by a typhoon. Damage to a set net and forestry.	Hamanaka Town (2015)

Sawai et al. Supplementary Table S4, *continued* . Historical and recent storms around the study area.

Date (Gregorian calendar)	Date (Japanese calendar)	Description	Reference
7-9 September 2004	Heisei 16th year, 9th month, 7th-9th days	Strong wind and waves by a typhoon. Damage to houses, fence, garages, cow sheds, a set net, etc.	Hamanaka Town (2015)
29-30 November 2005	Heisei 17th year, 11th month, 29th-30th days	Low pressure. Damage by strong wind to houses and cow sheds.	Hamanaka Town (2015)
7-8 October 2006	Heisei 18th year, 10th month, 7th-8th days	Low pressure. Sever damage to houses, agricultual, forestry, fishery facilities, and roads.	Hamanaka Town (2015)
6-8 January 2007	Heisei 19th year, 1st month, 6th-8th days	Low pressure. Damage to agricultua facility, commercial garages, etc.	Hamanaka Town (2015)
6 September 2007	Heisei 19th year, 9th month, 6th day	Damage by a typhoon to coastal roads.	Hamanaka Town (2015)
20 November 2007	Heisei 19th year, 11th month, 20th day	Damage by strong wind to houses and warehouses.	Hamanaka Town (2015)
1 April 2008	Heisei 20th year, 4th month, 1st day	Damage by strong wind and snow to houses and agricultual and fishery facilities.	Hamanaka Town (2015)
12 September 2008	Heisei 20th year, 9th month, 12th day	Heavy rain. Inundation below and above floor level.	Hamanaka Town (2015)
22 December 2010	Heisei 22nd year, 12th month, 22th day	Low pressure. Heavy rain. Inundation of town roads.	Hamanaka Town (2015)
22 September 2011	Heisei 23rd year, 9th month, 22th day	Strong wind by a typhoon. Dmage to houses, shops, a gym, fishin vessels.	Hamanaka Town (2015)
3 April 2012	Heisei 24th year, 4th month, 3rd day	Sediments transported by low pressure onto drying ground for seaweeds.	Hamanaka Town (2015)

References in supplementary data

Akkeshi Town. *Akkeshi Cho Shi* (Akkeshi Town, 1975) (in Japanese)

Hamanaka Town. *Shin Hamanaka Cho Shi* (Hamanka Town, 2015) (in Japanese)

Hendey, N. I. *An introductory account of the smaller algae of British coastal waters. V. Bacillariophyceae (Diatoms).* (Otto Koeltz Scientific Publishers, 1964).

Kawashima, A. & Mayama, S. Diatoms from Akan-ko (Lake Akan) in Hokkaido, Japan. 7. Raphid diatoms: Caloneis, Pinnularia. *Natural Environmental Science Research* **13**, 67–83 (2000). (in Japanese with English abstract)

Kobayasi, H. *et al. H. Kobayasi's Atlas of Japanese Diatoms Based on Electron Microscopy 1* (Uchida Rokakuho, 2006).

Krammer, K. & Lange-Bertalot, H. *Suswasserflora von Mitteleuropa. Bacillariophyceae 1. Teil:Naviculaceae.* (Gustav Fischer Verlag, 1986).

Krammer, K. & Lange-Bertalot, H. *Suswasserflora von Mitteleuropa. Bacillariophyceae 2. Teil:Bacillariaceae, Epithemiaceae, Surirellaceae.* (Gustav Fischer Verlag, 1988).

Krammer, K. *Diatoms of Europe. The genus Pinnularia.* (A.R.G. Gantner Verlag K.G., 2000)

Krammer, K. & Lange-Bertalot, H. *Suswasserflora von Mitteleuropa. Bacillariophyceae 3. Teil:Centrales, Fragilariaceae, Eunotiaceae.* (Gustav Fischer Verlag, 1991).

Patrick, R. & Reimer, C. *The Diatoms of United States. Exclusive of Alaska and Hawaii. Volume 1.* (The Academy of Natural Sciences of Philadelphia, 1966).

Patrick, R. & Reimer, C. *The Diatoms of United States. Exclusive of Alaska and Hawaii. Volume 2.* (The Academy of Natural Sciences of Philadelphia, 1975).

Round, F.E. *et al*. *The diatoms. Biology and morphology of the genera*. (Cambridge University Press, 1990).

Sawai, Y. & Nagumo, T. Diatom (Bacillariophyceae) flora of salt marshes along the Pacific coast of eastern Hokkaido, northern Japan. *Bulletin of The Nippon Dental University General Education* **32**, 93–108 (2003).

Sawai, Y. *et al.* Relationships between diatoms and tidal environments in Oregon and Washington, USA. *Diatom Research* **31**, 17–38 (2016).

Vos, P. C. & De Wolf, H. Reconstruction of sedimentary environments in Holocene coastal deposits of the southwest Netherlands; the Poortvliet boring, a case study of palaeo-environmental diatom research. *Hydrobiologia* **269–270**, 297–306 (1993).
